# Implementation of guidelines for multidisciplinary team management of pregnancy in women with pre-existing diabetes or cardiac conditions: results from a UK national survey

**DOI:** 10.1186/s12884-017-1609-9

**Published:** 2017-12-22

**Authors:** Cath Taylor, David R. McCance, Lucy Chappell, Catherine Nelson-Piercy, Sara A. Thorne, Khaled M. K. Ismail, James S. A. Green, Debra Bick

**Affiliations:** 1 University of Surrey, Faculty of Health and Medical Sciences, School of Health Sciences, Guildford, GU2 7XH UK; 2 Regional Centre for Endocrinology and Diabetes, Royal Victoria Hospital, Grosvenor Road, Belfast, BT12 6BA UK; 3 King’s College London, Women’s Health Academic Centre, Division of Women’s Health, London, SE1 7EH UK; 4 Department of Cardiology, Queen Elizabeth Hospital NHS Foundation Trust, Birmingham, B15 2TH UK; 5 The Birmingham Centre of Women’s and Children’s Health, College of Medical and Dental Sciences, University of Birmingham, Birmingham, B15 2TT UK; 6 Whipps Cross Hospital, Barts Health NHS Trust, London, UK; 7 Department of Health and Social Care, London South Bank University, London, UK; 8 King’s College London, Florence Nightingale Faculty of Nursing & Midwifery, London, SE1 8WA UK

**Keywords:** Diabetes, Cardiac conditions, Multidisciplinary care, Pregnancy

## Abstract

**Abstract:**

**Background:**

Despite numerous publications stating the importance of multidisciplinary care for women with pre-existing medical conditions, there is a lack of evidence regarding structure or processes of multidisciplinary working, nor impact on maternal or infant outcomes. This study aimed to evaluate the implementation of guidelines for multidisciplinary team (MDT) management in pregnant women with pre-existing diabetes or cardiac conditions. These conditions were selected as exemplars of increasingly common medical conditions in pregnancy for which MDT management is recommended to prevent or reduce adverse maternal and fetal outcomes.

**Methods:**

National on-line survey sent to clinicians responsible for management or referral of women with pre-existing diabetes or cardiac conditions in UK National Health Service (NHS) maternity units. The survey comprised questions regarding the organisation of MDT management for women with pre-existing diabetes or cardiac conditions. Content was informed by national guidance.

**Results:**

One hundred seventy-nine responses were received, covering all health regions in England (162 responses) and 17 responses from Scotland, Wales and Northern Ireland. 132 (74%) related to women with diabetes and 123 (69%) to women with cardiac conditions. MDT referral was reportedly standard practice in most hospitals, particularly for women with pre-existing diabetes (88% of responses vs. 63% for cardiac) but there was wide variation in relation to MDT membership, timing of referral and working practices. These inconsistencies were evident within and between maternity units across the UK. Reported membership was medically dominated and often in the absence of midwifery/nursing and other allied health professionals. Less than half of MDTs for women with diabetes met the recommendations for membership in national guidance, and although two thirds of MDTs for women with cardiac disease met the core recommendations for membership, most did not report having the extended members: midwives, neonatologists or intensivists.

**Conclusions:**

The wide diversity of organisational management for women with pre-existing diabetes or cardiac conditions is of concern and merits more detailed inquiry. Evidence is also required to support and better define the recommendations for MDT care.

**Electronic supplementary material:**

The online version of this article (10.1186/s12884-017-1609-9) contains supplementary material, which is available to authorized users.

## Background

Having a pre-existing maternal medical condition is a key risk factor for adverse pregnancy outcomes for mother and baby. Indeed the review of maternal deaths in the UK during 2009–2013 [1] found that indirect causes (exacerbation or new onset of medical or psychiatric disease) accounted for two thirds of maternal deaths during or after pregnancy. Two medical conditions that are increasingly common in pregnancy are diabetes and cardiac disease. Between 0.2–2% of pregnant women in the UK have pre-existing diabetes [2] and 1% are affected by heart disease [3]. These pregnancies are associated with increased risks of adverse outcomes for both mother and baby [1, 2, 4–8].

In the UK, National Institute for Health and Care Excellence (NICE) guidelines recommend women with pre-existing diabetes are referred immediately once pregnant to a ‘joint diabetes and antenatal clinic’ [9] and a National Enquiry into diabetes in pregnancy recommended the minimum team composition (*obstetrician, diabetes physician, diabetes specialist nurse, diabetes midwife and dietician)* [2]. Similarly, numerous publications recommend MDT management for women with pre-existing cardiac conditions [5, 7, 10]. The European Society of Cardiology published consensus guidelines recommending that ‘high-risk patients should be treated by an MDT in specialised centres’ [7], and the Royal College of Obstetricians and Gynaecologists (RCOG) recommend all women are at least initially referred for risk assessment by a core MDT including an obstetrician, cardiologist and anaesthetist [5] (with midwives, neonatologists and intensivists involved when appropriate) [11]. Similar recommendations for multidisciplinary management appear in guidelines globally ([6]). However, implementation of guidance has not been audited, nor does the guidance specify how these MDTs should be operationalised (e.g. leadership, mode/frequency of meeting with each other and with women and their partners, pathways into and out of the MDT). Furthermore, a systematic review by the authors found no critical evaluation of MDT models or impact on maternal or infant outcomes [12].

Consequently, the objectives of this audit were to evaluate the implementation of UK recommendations for managing pregnancy in women with pre-existing diabetes or cardiac conditions, and to describe and compare current service provision.

## Methods

### Sample and setting

An online UK survey aimed to achieve geographical representation by targeting senior specialists involved in referring or managing pregnant women with either pre-existing diabetes and/or cardiac conditions. There is no single data source to ascertain these senior specialists, so national organisations were approached who agreed to distribute the survey link to their members. The organisations included: British Maternal and Fetal Medicine Society (BMFMS); Royal College of Obstetricians and Gynaecologists (RCOG) Clinical Directors’ members; National Institute for Health Research (NIHR) Diabetes in Pregnancy Network (subgroup of the Diabetes Research Network); McDonald UK Obstetric Medicine Society (MOMS); NIHR Reproductive Health and Childbirth Research Network; and NIHR Cardiovascular Research Network. In addition, authors circulated the invitation to their networks of colleagues.

In the UK, maternity care is mostly provided in NHS hospitals that either serve their local population only (secondary care) or also receive referrals from other hospitals (tertiary care). Hospitals are managed by NHS trusts in England (*N* = 139 trusts provide maternity care in England, within 10 health regions), and by unified Health Boards in Scotland (*N* = 14), Wales (*N* = 7) and Northern Ireland (*N* = 5). Health trusts/boards vary in size and may include one or more hospitals with one or more maternity units.

### Survey

Respondents were screened to confirm they either referred or managed pregnant women with pre-existing cardiac conditions and/or diabetes. The survey comprised: background information (professional discipline; geographic location and type of unit - secondary/tertiary provider); and details of MDT management for women with pre-existing a) cardiac conditions; b) Type 1 or 2 diabetes. Piloting with five volunteer obstetricians highlighted the range of care defined as multidisciplinary teamwork (from specialists working in parallel with limited or ad-hoc direct communication, through to joint clinics where specialists met together with the women). The stem question and response options about MDT management were therefore designed to capture this variation, and based upon a framework distinguishing degree of integration between specialists [13] (Table 4).

Respondents who stated their current practice was referral to an MDT were asked about team membership, whether the team met in clinic with the pregnant women and/or separate to clinic (e.g. as a clinical team without the woman present), and typical timing of first referral to the MDT. Those who stated they referred to a ‘link’ clinician were asked the discipline and timing of referral. Those having ‘no formalised procedure’ or selecting ‘other’ were asked to describe the disciplines involved, the typical timing of referral, and any variation in practice. The full survey is available as an online link (see Additional file 1).

### Analysis

Data were imported into IBM SPSS v.22 for analysis. Responses were checked for completeness and eligibility (e.g. removing those not responsible for referral or management of either cardiac conditions or diabetes; and non-UK respondents). Multiple responses (between two and six) were received for some English trusts (*n* = 37), and their concordance was examined in relation to the overall ‘model’ of care they reported, and subsequent responses (i.e. membership, timing or referral). For the management of diabetes, responses in relation to all 25 trusts for which multiple responses were received were discordant, either in relation to the type of MDT model or details of the MDT model. For cardiac conditions, responses for 26 of the 29 trusts that had multiple responses were discordant. As data were at Trust/Board level (not hospital/unit), all responses were included as independent. Data were filtered by condition (diabetes/cardiac disease) and organisational model (as per Table 4) and analysed descriptively. Team composition for women with diabetes was evaluated against the recommendation that “*as a minimum the MDT should include an obstetrician, diabetes physician, diabetes specialist nurse, diabetes midwife and dietician*” [2]. Responses were coded as meeting this recommendation if the team included: any obstetrician (including those with or without Advanced Training qualifications); a diabetes specialist nurse; a diabetes midwife; a dietician and either a diabetologist or endocrinologist. In the UK there are usually two types of specialist dealing with diabetes: a) diabetologists who are general physicians with specialist interests in Diabetes (usually located in secondary care, District General Hospitals); b) endocrinologists who are specialists in endocrinology and/or diabetes (with less general medicine input), usually located in regional (tertiary) centres and oversee management of more complex patients.

For women with cardiac conditions, recommendations for core (obstetrician, cardiologist, anaesthetist), and extended (midwife, intensivist, neonatologist) membership [5, 11] were similarly assessed.

## Results

### Characteristics of the sample

A total of 179 responses were received (Table 1), over half from obstetricians (table 2).

**Table 1:** Source of survey responses

		Number of responses	% of total responses
BMFMS: British Maternal & Fetal Medicine Society	The BMFMS aims to improve the standard of pregnancy care by dissemination knowledge, promoting and funding research, contributing to the development and implementation of high quality training, and providing a forum where issues relevant to pregnancy care are discussed. http://www.bmfms.co.uk/	46	25.7
NIHR Cardiovascular Research Network	National Community of clinical practice (clinicians and researchers with local and national expertise). http://www.crn.nihr.ac.uk/cardiovascular/	8	4.5
NIHR Diabetes Research Network	National Community of clinical practice (clinicians and researchers with local and national expertise). http://www.crn.nihr.ac.uk/diabetes/	65	36.3
MOMS: MacDonald UK Maternal Obstetric Medicine Society	Support doctors who are interested in specialising in Obstetric or Maternal Medicine and provide a resource for generalists who are asked to advice pregnant women with medical conditions. http://www.obstetricmedic.org.uk/	22	12.3
NIHR Reproductive Health and Childbirth Research Network	National Community of clinical practice (clinicians and researchers with local and national expertise). http://www.crn.nihr.ac.uk/reproductivehealth/	2	1.1
RCOG: Royal College of Obstetricians and Gynaecologists	Works to improve women’s health worldwide. Over 12,500 members including fellows and affiliates.	16	8.9
Other direct contacts	Colleagues (including clinical directors) known to the authors	20	11.2
Total		179	100.0

**Table 2:** Respondents to the questionnaire by professional grouping

Professional Group	Number	%
Obstetrician	102	57.0
Diabetologist/Endocrinologist	18	10.1
Diabetes specialist midwife	13	7.3
Diabetes specialist nurse	11	6.1
Anaesthetist	11	6.1
Midwife	9	5.0
Cardiologist	8	4.5
Dietician	3	1.7
Obstetric Physician	3	1.7
Intensivist	1	.6
Total	179	100.0

Two thirds of respondents (120, 67%) worked in secondary provider settings, and a third (59, 33%) in a tertiary setting. Responses from England covered 92 (67%) of the 139 NHS trusts providing maternity care, and included all health regions. There were two responses from Northern Ireland, three from Wales and 12 from Scotland (Table 3).

**Table 3:** Geographical spread of responses

	Number of responses	%	Overall (*N* Trusts with at least one response/Total N Trusts in region^b^)	Diabetes (*N* Trusts with at least one response)	Cardiac (*N* Trusts at least one response)
ENGLAND					
London	27	15.2	15/22	13	13
South West	23	12.9	12/16	10	8
South Central	10	5.6	5/9	5	4
South East Coast	8	4.5	6/11	6	2
East England	16	9.0	11/17	9	5
Yorkshire & Humber	15	8.4	9/13	8	8
West Midlands	21	11.8	13/15	10	13
East Midlands	8	4.5	5/8	3	5
North East	16	9.0	6/8	4	6
North West	17	9.6	10/20	8	8
Total number in England	161	90.5	92/139	76	72
SCOTLAND	12	6.7	12 responses covering ≥7/14 health boards *(2 responses only identified as “Scotland”)*	6	6
WALES	3	1.7	3 responses covering 3 of the 7 local health boards.	2	3
NORTHERN IRELAND	2	1.1	2 responses covering 2 of the 5 health and social care Trusts	2	2
Total	178^a^	100.0			

^a^ *Number of Trusts in region taken from HSCIC maternity service provider report*

^b^ *Trust name missing for one respondent*

### Management of pregnant women with congenital or acquired cardiac disease

123 (69%) respondents stated they either referred or managed pregnant women with congenital or acquired cardiac disease. Responses covered all UK regions in similar proportions to the overall pattern of responses (Table 3).

Two thirds of respondents stated that such women would be managed by an MDT, either in a tertiary (38%) or secondary (24%) setting (Table 4). A fifth of respondents stated they referred to a named link/specialist clinician (46% referring to a cardiologist; 29% to an obstetrician with advanced training; 17% to an obstetrician without such training), and 15 (12%) replied that they had no formalized procedures in place. Five (4%) selected ‘other organisational model’. Those with no formalized procedures or ‘other’ models described a range of models and membership including letter/email referrals to non-specific individuals on an ad-hoc basis; referral to a separate anaesthetic clinic; and “close liaison with the local cardiologist”. There was no regional pattern in responses; management by tertiary or secondary care MDTs was stated in all regions. Responses stating they had ‘no formalized procedure’ came from trusts within eight regions in England, and two health boards in Scotland and Northern Ireland.

**Table 4:** Organisational models for antenatal management

Which of the following best describes the way that decisions are reached about the management of women with pre-existing cardiac conditions or diabetes antenatally? Select the option that best reflects your current practice:	Cardiac conditions		Diabetes Type1/2	
	Number	%	Number	%
Referral directly to a specialist MDT in a tertiary centre *(A multidisciplinary team of clinicians and midwives with different expertise who meet – either face to face or using videoconferencing – regularly to discuss individual cases – either in clinic or other setting)*	47	38.2	42	31.8
Referral to a local (secondary care based) MDT with relevant expertise at least in the first instance *(then perhaps subsequent referral to a specialist tertiary team if deemed necessary)*	30	24.4	74	56.1
Referral to a named link/specialist clinician/individual	26	21.1	16	12.1
No formalized procedures in place or named link individuals. Referrals made on an ad-hoc basis.	15	12.2	0	0
Other organisational model	5	4.1	0	0
Total	123	100.0	132	100.0

NB: 15 responses regarding cardiac care (9 having a specialist MDT and 6 secondary care MDT), and 2 responses regarding diabetes care (1 specialist MDT and 1 secondary care MDT) selected an option but did not answer subsequent questions. These responses are included above but denominators will be different in other tables due to this missing data

### MDT cardiac models

#### Membership

Membership of tertiary cardiac MDTs ranged from 2 to 7 (average 4 members); Membership of secondary MDTs ranged from 2 to 6 (average 3 members). The most commonly reported members were cardiologists, anaesthetists and obstetricians, two thirds had all three members as per the core membership guidelines [11] (Table 5). Only one tertiary team (and no secondary teams) reported also having the three recommended ‘extended’ members: Midwife, Intensivist and Neonatologist. All three were absent in 21 teams (20% tertiary and 40% secondary MDTs). A number of other disciplines were listed as team members by a minority of respondents (Table 5).

**Table 5:** Membership of tertiary and secondary MDTs for cardiac conditions

	Tertiary MDT (*N* = 47)		Secondary MDT (*N* = 30)	
Professional Group	Number	%	Number	%
Cardiologist	36	76	15	50
Obstetrician (Advanced or sub-specialist trained in maternal medicine)	31	66	11	37
Anaesthetist	30	64	14	47
Midwife:				
Specialist cardiac midwife	11	23	3	10
Woman’s named midwife	3	6	1	3
Other midwife	17	36	6	20
Fetal cardiologist	14	30	3	10
Obstetric Physician	9	19	5	17
Obstetrician	9	19	8	27
Specialist nurse	4	9	1	3
Neonatologist	4	9	3	10
GP	1	2	0	0
Intensivist	1	2	0	0
Other physician	1	2	4	13
Other GUCH consultant; Cardiology technicians; fetal medicine midwives, haematologists	4	9	0	0

#### Mode of working

Most MDTs (tertiary and secondary care) met within the clinic setting only (64% and 82% respectively). Some tertiary MDTs (8, 22%) and secondary MDTs (10, 14%) met as a team both in the clinic and separately. However a minority of tertiary MDTs (5, 14%) and secondary MDTs (3, 4%) only met separately to the clinic setting.

#### Timing of referral

Most women were referred to MDTs either at first contact with health services when pregnant (e.g. when visiting GP/family doctor to confirm pregnancy) or at the first hospital-based antenatal booking visit. However, in some units referral did not occur until first contact with the medical lead, or following the 18–20 week routine anomaly scan (Table 6).

**Table 6:** Timing of referral to tertiary MDT, secondary MDT or named link clinician

Once pregnant, at what point during a women’s pregnancy is the first referral usually made to the MDT?						
	Cardiac conditions *N* (%)			Diabetes *N* (%)		
	Tertiary MDT	Secondary MDT	Named Link clinician	Tertiary MDT	Secondary MDT	Named link clinician
First contact with health services when pregnant *(*i.e. *GP pre-booking visit)*	15 (40)	6 (25)	7 (29)	33 (81)	65 (89)	11 (73)
Booking visit (8–12 weeks)	13 (34)	12 (50)	8(33)	8 (20)	5 (7)	2 (13)
First scan (12 week scan)	1 (3)	5 (21)	2(8)			
First hospital appointment with medical lead for the condition	4 (11)	0	6(25)			1 (7)
Anomaly scan (18–20 weeks)	0	1 (4)	0			
Other (please describe)	3 (8): varies according to complexity of condition. Some women self-refer.		1 (4): at any point between booking and delivery with obstetricians decide to refer)		3 (4) variable depending on practice; patients can self-refer and usually seen same day; referrals from CMW, GP, DSN and self-referral often 4–8 weeks, occasionally 8–12 weeks	1 (7) ad hoc, sometimes community midwife refers at booking or GP routine referral

### Management of pregnant women with type 1 or 2 diabetes mellitus

132 (74%) respondents stated they referred or managed pregnant women with type 1 or 2 diabetes (Table 3). Most (116, 88%) stated that such women were managed by an MDT, either in a tertiary (32%) or secondary (56%) setting (Table 4). A minority (12%) reported referral to a link specialist clinician instead of an MDT, including diabetologists (*n* = 4), obstetricians (with advanced training *n* = 4; no advanced training *n* = 3), specialist diabetes midwives (*n* = 3) and obstetric physician (*n* = 1). One respondent stated that women were referred to a uni-disciplinary “diabetes or obstetric team”. There was no discernible geographic pattern: all regions reported both tertiary and secondary MDT models, and “named link specialist” models were reported in seven health regions in England and one health board in Scotland.

### MDT diabetes models

#### Membership

Tertiary MDTs reported between 4 and 9 members (average 6), and secondary MDTs between 3 and 8 members (average 5). Less than half of all MDTs (18/41, 44% tertiary MDTs; 36/73, 49% secondary MDTs) had all five “minimum membership” specialists represented [2]. All MDTs included a diabetologist or endocrinologist, but most lacked at least one other specialist, particularly dieticians, specialist nurses and specialist midwives (though four tertiary and three secondary teams also lacked obstetric input). Other specialists represented in a small number of MDTs included anaesthetists, GPs, obstetric physicians, intensivists and neonatologists (Table 7).

**Table 7:** Membership of tertiary and secondary MDTs for diabetes

	Tertiary MDT (*N* = 42)		Secondary MDT (*N* = 74)	
Professional Group	*N*	%	*N*	%
Diabetologist	42	100	65	88
Endocrinologist	12	29	24	32
Obstetrician (with advanced training)	33	79	37	50
Dietician	33	79	62	84
Midwife:				
Specialist diabetes midwife	35	83	50	68
Woman’s named midwife	1	2	5	7
Other midwife	18	43	17	23
Specialist diabetes nurse	35	83	64	86
Obstetric Physician	10	24	6	8
Obstetrician (without advanced training)	12	29	41	55
Anaesthetist	7	17	2	3
Neonatologist	1	2	1	1
GP	1	2	1	1
Other – *Specialist: healthcare assistant* *Secondary: Administrative support and sonographer/ Assistant practitioner for diabetes*	1	2	2	3

#### Mode of working

Most MDTs (tertiary and secondary care) met within the clinic setting only (74% and 80% respectively). Some tertiary MDTs (8, 19%) and secondary MDTs (4, 5%) met both in clinic and separately. A minority of tertiary MDTs (2, 5%) and secondary MDTs (4, 5%) only met separately to the clinic.

#### Timing of referral

All referrals to MDTs occurred either at first contact with health services when pregnant or at the first hospital-based antenatal booking visit.

## Discussion

Recommendations for MDT care during pregnancy for women with pre-existing diabetes or cardiac conditions have been implemented inconsistently across the UK. Although some form of MDT referral was standard practice in many units, the survey revealed wide variation in relation to membership, timing of referral and working practices. These inconsistencies were evident both within and between different trusts and regions of the UK.

For women with pre-existing cardiac conditions, a third of respondents (covering 47 UK units) stated that referrals were not to an MDT and instead to an individual “link” clinician, or there was ‘no formalized procedure’ of referral in place. Furthermore, in units where referral was to an MDT, the membership was typically medically dominated and often without midwifery/nursing and other extended membership particularly neonatologists and intensivists. Referral timing also varied; in some units not occurring until the fetal anomaly scan at 18–20 weeks gestation. For women with pre-existing diabetes, where NICE guidance recommends immediate referral once pregnant to a joint diabetes and obstetric team [9], most sites had MDTs, and referral was early in pregnancy. However, less than half of the MDTs comprised the ‘minimum’ recommended membership [2], most frequently omitting a dietician, specialist nurse and/or specialist midwife. Furthermore, a minority of MDTs only met separately to the clinic setting (and therefore by inference were not providing a joint clinic), and in a few units referral was to an individual specialist.

The importance of multi-professional working to safe and effective maternity care is further emphasised by the recent National Maternity Review [14]. The omission of midwives and nurses from MDTs is of concern, but perhaps unsurprising given the recognised shortage – and projections of further decline - of NHS staff including midwives and nurses [15]. Whilst a recent study of the maternity workforce in England [16] found that increasing the number of obstetricians had the greatest impact on outcomes in high-risk women, this should be balanced against the critical role of MDT management of such women. MDT input from midwives and specialist nurses in particular is necessary to promote recovery, support breastfeeding, and provide advice on healthy lifestyle behaviours. Such extended MDT support to inform life-course health could have considerable benefit [17].

To our knowledge this is the first UK (or indeed global) study examining the organisation of care for women with pre-existing medical conditions in pregnancy. The survey design was informed by a framework of ‘degree of integration’ of healthcare [13], and team membership was assessed against existing guidelines. Due to limited resources we could not send reminders, which may have increased the response rate and thereby the generalisability of findings. However the responses represented two thirds of trusts in England and included representation from health boards in Scotland, Northern Ireland and Wales. Our findings are limited to provider-level interpretation as this was the only identifier in the dataset. If repeated it would be beneficial to include a unit/hospital level identifier to explore more fully variation within healthcare organisations as well as between them. The audit relied on self-reported data from one respondent in each site (in most cases). The lack of a single database of UK clinical leads for these medical conditions meant that it was necessary to seek the assistance of a range of organisations to distribute the survey to relevant professional members. However all included respondents confirmed they were responsible for either referring or managing women with cardiac conditions and/or diabetes. Data were not validated or checked for accuracy against practice and it is possible that some survey responses contained inaccuracies.

The diversity in practice uncovered is perhaps not surprising given the lack of guidance about operationalising multidisciplinary care for these conditions, and may also reflect limited resources. This differs from UK cancer care where comprehensive guidelines exist regarding team structure (at local, regional and national levels) (https://www.nice.org.uk/guidancemenu/conditions-and-diseases/cancer), and a national peer review programme ensures links to NHS commissioning. MDTs in cancer have been associated with better patient care [18, 19] but evidence to support MDTs in maternal medicine is lacking [12]. While there may be a number of explanations, including economic reasons, for the diversity in the models of care these may have important short and long term clinical implications for both mother and baby.

Further research is needed to identify the key elements of clinically (and cost) effective models of care before, during and after pregnancy for women with pre-existing medical conditions. Effectiveness should be considered in relation to outcomes for the women (including clinical outcomes and experience of care), the infant, the team, and wider organisation, and should take account of the different contexts and geographical settings in which maternity care is provided. Recent findings from the UK National Diabetes in Pregnancy Audit [20] show there is still much to be done to improve outcomes. The impact of the diversity of MDT management on outcomes is unknown and should be a priority focus for future research.

## Conclusions

Despite current guidance and consensus opinion for the use of MDTs when caring for pregnant women with pre-existing medical conditions, there continues to be a lack of primary research to support the clinical and cost effectiveness of this approach to care or to define how such care should be implemented or evaluated. Life course health for women with serious medical conditions and their infants are compromised if pregnancy and birth are not optimally managed. If indirect causes of maternal death and maternal and fetal morbidity from medical disease in pregnancy are to be reduced, research is urgently needed to promote appropriate service provision, led by optimal MDTs which include clinicians with appropriate skills to provide evidence based care across the entire pregnancy pathway, including pre and post pregnancy. Without further research into composition, location and referral pathways, MDT care is likely to persist as ad-hoc and fragmented.

## Additional file

Additional file 1: Taylor Maternal Survey. The survey used in the study (PDF 414 kb)

## Abbreviations

BMFMS: British Maternal & Fetal Medicine Society
IBM: International Business Machines
MDT: Multidisciplinary Team
MOMS: Macdonald Obstetric Medicine Society
NHS: National Health Service
NICE: National Institute for Health and Care Excellence
NIHR: National Institute for Health Research
RCOG: Royal College of Obstetricians and Gynaecologists
SPSS: Statistical Package for the Social Sciences
UK: United Kingdom
