# Supplementary material for: Implementation of guidelines for multidisciplinary team management of pregnancy in women with pre-existing diabetes or cardiac conditions: results from a UK national survey
Source: BMC Pregnancy Childbirth. 2017 Dec 22;17:434. doi: 10.1186/s12884-017-1609-9 (PMC5741950; doi:10.1186/s12884-017-1609-9)
Supplement: Additional file 1: — Taylor Maternal Survey. The survey used in the study (PDF 414 kb) [file 12884_2017_1609_MOESM1_ESM.pdf]

Introduction

Many of us work in a multidisciplinary team of one sort or another and we are interested in finding out more about these teams, how they work, which bits work well, what we could learn from multidisciplinary teams in different primary and secondary care settings. We think that multidisciplinary teams could be a particularly important approach for looking after pregnant women with pre-existing medical conditions – who may be looked after by one team for their medical condition, and another for their pregnancy, with their midwife and GP involved to varying degrees.

This short questionnaire aims to collect information about models of multidisciplinary care used for pregnant women with **congenital or acquired cardiac disease/conditions and pre-existing Type 1 or Type 2 Diabetes Mellitus.** We will use this information to inform further research into the effectiveness of different models of care for women during and after pregnancy.

Completing this questionnaire should only take approximately 5mins. The closing date is Friday 8th March.

We are very grateful for your support.

Core Research team:

Professor Debra Bick, Kings College London

Dr Cath Taylor, Kings College London

Sarah Beake, Kings College London

Clinical members of research team:

Professor James Green, Barts Health NHS Trust

Professor Khaled Ismail, University of Birmingham

Dr Lucy Chappell, Kings College London

Mr Robert Sawdy, Poole Hospital and RBCH NHS Foundation Trusts

To contact us directly please email Cath.Taylor@kcl.ac.uk

Clinical role

These questions are about you and your clinical role

1. What is your professional group?

- Obstetrician
- Anesthetist
- Obstetric Physician
- General practice
- Intensivist
- Physician (please state clinical specialty)
- Midwife: (please state clinical title)
- Other

Please specify your professional group if you have selected *other*, your clinical speciality if you selected *physician* or your clinical title if you selected *midwife* above:

2. What is the name of the Trust/unit you work in? *(this information will be used to map responses as we aim to gain coverage across England)*

3. Which level of care does your usual workplace provide?

- Primary care (general practice)
- Secondary care (hospital)
- Tertiary unit (also taking referrals from other hospitals)

Other (please describe)

Management of pregnant women with cardiac conditions

4. Are you involved in the referral or management of pregnant women with cardiac conditions?

- ◯ Yes
- ◯ No

Management of pregnant women with congenital or acquired cardiac disease

5. Which of the following best describes the way that decisions are reached about the management of such women antenatally? Select the option that best reflects your current practice:

- ◯ A woman is referred directly to a specialist team in a tertiary centre: A multidisciplinary team of clinicians and midwives with different expertise who meet (either face to face or using videoconferencing) regularly to discuss individual cases –either in clinic or other setting.
- ◯ A woman is referred to a local (secondary care based) multidisciplinary team with relevant expertise at least in the first instance (then perhaps subsequent referral to a specialist tertiary team if deemed necessary)
- ◯ A woman is referred to a named link/specialist clinician/individual
- ◯ No formalized procedures in place or named link individuals. Referrals made on an ad-hoc basis.
- ◯ Other organisational model

Referral to a specialist team in a tertiary site

6. When was the specialist team first set up?

- ◯ Within the last 12 months
- ◯ 12-24 months ago
- ◯ Over 24 months ago
- ◯ Don't know

7. What is the membership of the specialist team? *Tick all that apply:*

- ☐ Cardiologist
- ☐ Obstetric Physician
- ☐ Other Physician (specify)
- ☐ Specialist cardiac midwife
- ☐ Woman’s named midwife
- ☐ Other midwife
- ☐ Specialist nurse
- ☐ Obstetrician (ATSM or sub specialist trained in maternal medicine)
- ☐ Obstetrician
- ☐ Neonatologist
- ☐ GP
- ☐ Anaesthetist
- ☐ Intensivist
- ☐ Fetal cardiologist
- ☐ Not sure
- ☐ Other

If you selected other above please specify:

____

8. How does the team meet? *Tick all that apply*

- ☐ Within the clinic setting
- ☐ Separate to the clinic (e.g. in an MDT meeting)

9. Approximately how many women with congenital or acquired cardiac conditions does the specialist team see for the first time <u>per month</u>?

- ◯ 1-2
- ◯ 3-5
- ◯ 6-10
- ◯ 11-20
- ◯ >20

10. Once pregnant, at what point during a woman's pregnancy is the first referral usually made to the MDT?

- ◯ First contact with health services when pregnant
- ◯ Booking visit (8-12 weeks)
- ◯ First scan (12 week scan)
- ◯ First hospital appointment with medical lead
- ◯ Anomaly scan (18-20 weeks)
- ◯ Other (please describe)

If you selected other above, please describe:

11. Do you have referral criteria in use for women with cardiac conditions?

- ◯ Yes
- ◯ No
- ◯ Don't know

12. Does regular evaluation of the outcomes of MDT care for women with cardiac conditions take place?

- ◯ Yes
- ◯ No
- ◯ Don't know

Referral to a local (secondary-care based) MDT

13. When was the team first set up?

- ◯ Within the last 12 months
- ◯ 12-24 months ago
- ◯ Over 24 months ago
- ◯ Don't know

14. What is the membership of the team? *Tick all that apply:*

- ☐ Cardiologist
- ☐ Obstetric Physician
- ☐ Other Physician (specify)
- ☐ Specialist cardiac midwife
- ☐ Woman's named midwife
- ☐ Other midwife
- ☐ Specialist nurse
- ☐ Obstetrician (ATSM or sub specialist trained in maternal medicine)
- ☐ Obstetrician
- ☐ Neonatologist
- ☐ GP
- ☐ Anaesthetist
- ☐ Intensivist
- ☐ Fetal cardiologist
- ☐ Not sure
- ☐ Other

If you selected other above please specify:

15. How does the team meet? *Tick all that apply*

- ☐ Within the clinic setting
- ☐ Separate to the clinic (e.g. in an MDT meeting)

16. Approximately how many women with congenital or acquired cardiac conditions does the specialist team see for the first time per month?

- ◯ 1-2
- ◯ 3-5
- ◯ 6-10
- ◯ 11-20
- ◯ >20

17. Once pregnant, at what point during a woman's pregnancy is the first referral usually made to the team?

- ◯ First contact with health services when pregnant
- ◯ Booking visit (8-12 weeks)
- ◯ First scan (12 week scan)
- ◯ First hospital appointment with medical lead
- ◯ Anomaly scan (18-20 weeks)
- ◯ Other (please describe)

If you selected other above, please describe:

18. Do you have referral criteria in use for women with cardiac conditions?

- ◯ Yes
- ◯ No
- ◯ Don't know

19. Does regular evaluation of the outcomes of MDT care for women with cardiac conditions take place?

- ◯ Yes
- ◯ No
- ◯ Don't know

20. Do you refer any women to a specialist MDT in a tertiary centre?

- ◯ Yes
- ◯ No

Referral to a specialist team in a tertiary site

21. When was the specialist team first set up?

- ◯ Within the last 12 months
- ◯ 12-24 months ago
- ◯ Over 24 months ago
- ◯ Don't know

22. What is the membership of the specialist team? *Tick all that apply:*

- ☐ Cardiologist
- ☐ Obstetric Physician
- ☐ Other Physician (specify)
- ☐ Specialist cardiac midwife
- ☐ Woman's named midwife
- ☐ Other midwife
- ☐ Specialist nurse
- ☐ Obstetrician (ATSM or sub specialist trained in maternal medicine)
- ☐ Obstetrician
- ☐ Neonatologist
- ☐ GP
- ☐ Anaesthetist
- ☐ Intensivist
- ☐ Fetal cardiologist
- ☐ Not sure
- ☐ Other

If you selected other above please specify:

23. How does the team meet? *Tick all that apply*

- ☐ Within the clinic setting
- ☐ Separate to the clinic (e.g. in an MDT meeting)

24. Approximately how many women with congenital or acquired cardiac conditions are referred to the specialist team for the first time <u>per month</u>?

- ◯ 1-2
- ◯ 3-5
- ◯ 6-10
- ◯ 11-20
- ◯ >20

25. Once pregnant, at what point during a woman's pregnancy is the first referral usually made to the MDT?

- ◯ First contact with health services when pregnant
- ◯ Booking visit (8-12 weeks)
- ◯ First scan (12 week scan)
- ◯ First hospital appointment with medical lead
- ◯ Anomaly scan (18-20 weeks)
- ◯ Other (please describe)

If you selected other above, please describe:

26. Do you have referral criteria in use for women with cardiac conditions?

- ◯ Yes
- ◯ No
- ◯ Don't know

27. Does regular evaluation of the outcomes of MDT care for women with cardiac conditions take place?

- ◯ Yes
- ◯ No
- ◯ Don't know

Referral to a named link/specialist clinician/individual

28. What is the professional group of the named link/specialist clinician/individual?

- ◯ Cardiologist
- ◯ Obstetric Physician
- ◯ Other Physician (specify)
- ◯ Specialist cardiac midwife
- ◯ Woman's named midwife
- ◯ Other midwife
- ◯ Specialist nurse
- ◯ Obstetrician (ATSM or sub specialist trained in maternal medicine)
- ◯ Obstetrician
- ◯ Neonatologist
- ◯ GP
- ◯ Anaesthetist
- ◯ Intensivist
- ◯ Fetal cardiologist
- ◯ Not sure
- ◯ Other

If you selected other above please specify:

____

29. Approximately how many women with congenital or acquired cardiac conditions are referred to this link person for the first time <u>per month</u>?

- ◯ 1-2
- ◯ 3-5
- ◯ 6-10
- ◯ 11-20
- ◯ >20

30. Once pregnant, at what point during a woman's pregnancy is the first referral usually made to the link person?

- ◯ First contact with health services when pregnant
- ◯ Booking visit (8-12 weeks)
- ◯ First scan (12 week scan)
- ◯ First hospital appointment with medical lead
- ◯ Anomaly scan (18-20 weeks)
- ◯ Other (please describe)

If you selected other above, please describe:

No formalized procedures in place or named link individuals. Referrals mad...

31. Please describe how decisions about the management of women with congenital or acquired cardiac conditions are usually made (in terms of the professionals involved and referral pathways, or variation in practice within your unit between different obstetricians etc).

Professionals involved and how they typically refer to each other:

Variation in practice within the unit (if applicable):

32. Approximately how many women with congenital or acquired cardiac conditions are referred for the first time per month?

- ◯ 1-2
- ◯ 3-5
- ◯ 6-10
- ◯ 11-20
- ◯ >20

Other organisational model

33. Please describe how decisions about the management of women with congenital or acquired cardiac conditions are usually made (in terms of the professionals involved and referral pathways, or variation in practice within your unit between different obstetricians etc).

Professionals involved and how they typically refer to each other:

Variation in practice within the unit (if applicable):

34. Approximately how many women with congenital or acquired cardiac conditions are referred for the first time <u>per month</u>?

- ◯ 1-2
- ◯ 3-5
- ◯ 6-10
- ◯ 11-20
- ◯ >20

Management of pregnant women with pre-existing Diabetes Mellitus

35. Are you involved in the referral or management of pregnant women with pre-existing Diabetes Mellitus?

- ◯ Yes
- ◯ No

Management of pregnant women with pre-existing Diabetes Mellitus

36. Which of the following best describes the way that decisions are reached about the antenatal management of women with pre-existing diabetes? Select the option that best reflects your current practice:

◯ A woman is referred directly to a specialist team in a tertiary centre: A multidisciplinary team of clinicians and midwives with different expertise who meet (either face to face or using videoconferencing) regularly to discuss individual cases –either in clinic or other setting.

◯ A woman is referred to a local (secondary care based) multidisciplinary team with relevant expertise at least in the first instance (then perhaps subsequent referral to a specialist tertiary team if deemed necessary)

◯ A woman is referred to a named link/specialist clinician/individual

◯ No formalized procedures in place or named link individuals. Referrals made on an ad-hoc basis.

◯ Other organisational model

◯ Women with Type 1 and Type 2 diabetes are managed using different organisational models (will allow you to answer this separately for each condition).

Referral to a specialist team in a tertiary site

37. When was the specialist team first set up?

- ◯ Within the last 12 months
- ◯ 12-24 months ago
- ◯ Over 24 months ago
- ◯ Don't know

38. What is the membership of the specialist team? *Tick all that apply:*

- ☐ Diabetologist
- ☐ Obstetric Physician
- ☐ Other Physician (specify)
- ☐ Specialist diabetes midwife
- ☐ Woman’s named midwife
- ☐ Other midwife
- ☐ Specialist diabetes nurse
- ☐ Obstetrician (ATSM or sub specialist trained in maternal medicine)
- ☐ Obstetrician
- ☐ Endocrinologist
- ☐ Neonatologist
- ☐ GP
- ☐ Anaesthetist
- ☐ Intensivist
- ☐ Dietician
- ☐ Not sure
- ☐ Other

If you selected other above please specify:

39. How does the team meet? *Tick all that apply*

- ☐ Within the clinic setting
- ☐ Separate to the clinic (e.g. in an MDT meeting)

40. Approximately how many women with pre-existing diabetes are does the specialist team see for the first time per month?

◯ 1-2

◯ 3-5

◯ 6-10

◯ 11-20

◯ >20

41. Once pregnant, at what point during a woman's pregnancy is the first referral usually made to the MDT?

◯ First contact with health services when pregnant

◯ Booking visit (8-12 weeks)

◯ First scan (12 week scan)

◯ First hospital appointment with medical lead

◯ Anomaly scan (18-20 weeks)

◯ Other (please describe)

If you selected other above, please describe:

42. Do you have referral criteria in use for women with pre-existing diabetes?

◯ Yes

◯ No

◯ Don't know

43. Does regular evaluation of the outcomes of MDT care for women with pre-existing diabetes take place?

◯ Yes

◯ No

◯ Don't know

Referral to a local (secondary-care based) MDT

44. When was the team first set up?

- ◯ Within the last 12 months
- ◯ 12-24 months ago
- ◯ Over 24 months ago
- ◯ Don't know

45. What is the membership of the team? *Tick all that apply:*

- ☐ Diabetologist
- ☐ Obstetric Physician
- ☐ Other Physician (specify)
- ☐ Specialist diabetes midwife
- ☐ Woman's named midwife
- ☐ Other midwife
- ☐ Specialist diabetes nurse
- ☐ Obstetrician (ATSM or sub specialist trained in maternal medicine)
- ☐ Obstetrician
- ☐ Endocrinologist
- ☐ Neonatologist
- ☐ GP
- ☐ Anaesthetist
- ☐ Intensivist
- ☐ Dietician
- ☐ Not sure
- ☐ Other

If you selected other above please specify:

____

46. How does the team meet? *Tick all that apply*

- ☐ Within the clinic setting
- ☐ Separate to the clinic (e.g. in an MDT meeting)

47. Approximately how many women with pre-existing diabetes does the specialist team see for the first time per month?

- ◯ 1-2
- ◯ 3-5
- ◯ 6-10
- ◯ 11-20
- ◯ >20

48. Once pregnant, at what point during a woman's pregnancy is the first referral usually made to the team?

- ◯ First contact with health services when pregnant
- ◯ Booking visit (8-12 weeks)
- ◯ First scan (12 week scan)
- ◯ First hospital appointment with medical lead
- ◯ Anomaly scan (18-20 weeks)
- ◯ Other (please describe)

If you selected other above, please describe:

49. Do you have referral criteria in use for women with pre-existing diabetes?

- ◯ Yes
- ◯ No
- ◯ Don't know

50. Does regular evaluation of the outcomes of local MDT care for women with pre-existing diabetes take place?

- ◯ Yes
- ◯ No
- ◯ Don't know

51. Do you refer any women to a specialist MDT in a tertiary centre?

- ◯ Yes
- ◯ No

Referral to a specialist team in a tertiary site

52. When was the specialist team first set up?

- ○ Within the last 12 months
- ○ 12-24 months ago
- ○ Over 24 months ago
- ○ Don't know

53. What is the membership of the specialist team? *Tick all that apply:*

- ☐ Diabetologist
- ☐ Obstetric Physician
- ☐ Other Physician (specify)
- ☐ Specialist diabetes midwife
- ☐ Woman's named midwife
- ☐ Other midwife
- ☐ Specialist diabetes nurse
- ☐ Obstetrician (ATSM or sub specialist trained in maternal medicine)
- ☐ Obstetrician
- ☐ Endocrinologist
- ☐ Neonatologist
- ☐ GP
- ☐ Anaesthetist
- ☐ Intensivist
- ☐ Dietician
- ☐ Not sure
- ☐ Other

If you selected other above please specify:

54. How does the team meet? *Tick all that apply*

- ☐ Within the clinic setting
- ☐ Separate to the clinic (e.g. in an MDT meeting)

55. Approximately how many women with pre-existing diabetes are referred to the specialist team for the first time <u>per month</u>?

- ◯ 1-2
- ◯ 3-5
- ◯ 6-10
- ◯ 11-20
- ◯ >20

56. Once pregnant, at what point during a woman's pregnancy is the first referral usually made to the MDT?

- ◯ First contact with health services when pregnant
- ◯ Booking visit (8-12 weeks)
- ◯ First scan (12 week scan)
- ◯ First hospital appointment with medical lead
- ◯ Anomaly scan (18-20 weeks)
- ◯ Other (please describe)

If you selected other above, please describe:

57. Do you have referral criteria in use for women with pre-existing diabetes?

- ◯ Yes
- ◯ No
- ◯ Don't know

58. Does regular evaluation of the outcomes of MDT care for women with pre-existing diabetes take place?

- ◯ Yes
- ◯ No
- ◯ Don't know

Referral to a named link/specialist clinician/individual

59. What is the professional group of the named link/specialist clinician/individual?

- ◯ Diabetologist
- ◯ Obstetric Physician
- ◯ Other Physician (specify)
- ◯ Specialist diabetes midwife
- ◯ Woman's named midwife
- ◯ Other midwife
- ◯ Specialist diabetes nurse
- ◯ Obstetrician (ATSM or sub specialist trained in maternal medicine)
- ◯ Obstetrician
- ◯ Endocrinologist
- ◯ Neonatologist
- ◯ GP
- ◯ Anaesthetist
- ◯ Intensivist
- ◯ Dietician
- ◯ Not sure
- ◯ Other

If you selected other above please specify:

____

60. Approximately how many women with pre-existing diabetes are referred to this link person for the first time per month?

- ◯ 1-2
- ◯ 3-5
- ◯ 6-10
- ◯ 11-20
- ◯ >20

61. Once pregnant, at what point during a woman's pregnancy is the first referral usually made to the link person?

- First contact with health services when pregnant
- Booking visit (8-12 weeks)
- First scan (12 week scan)
- First hospital appointment with medical lead
- Anomaly scan (18-20 weeks)
- Other (please describe)

If you selected other above, please describe:

No formalized procedures in place or named link individuals. Referrals mad...

62. Please describe how decisions about the management of women with pre-existing diabetes are usually made (in terms of the professionals involved and referral pathways, or variation in practice within your unit between different obstetricians etc).

Professionals involved and how they typically refer to each other:

Variation in practice within the unit (if applicable):

63. Approximately how many women with pre-existing diabetes are referred for the first time <u>per month</u>?

- ◯ 1-2
- ◯ 3-5
- ◯ 6-10
- ◯ 11-20
- ◯ >20

Other organisational model

64. Please describe how decisions about the management of women with congenital or acquired cardiac conditions are usually made (in terms of the professionals involved and referral pathways, or variation in practice within your unit between different obstetricians etc).

Professionals involved and how they typically refer to each other:

Variation in practice within the unit (if applicable):

65. Approximately how many women with congenital or acquired cardiac conditions are referred for the first time per month?

- 1-2
- 3-5
- 6-10
- 11-20
- >20

66. Which of the following best describes the way that decisions are reached about the antenatal management of women with pre-existing Type 1 diabetes? Select the option that best reflects your current practice:

- ◯ A woman is referred directly to a specialist team in a tertiary centre: A multidisciplinary team of clinicians and midwives with different expertise who meet (either face to face or using videoconferencing) regularly to discuss individual cases –either in clinic or other setting.
- ◯ A woman is referred to a local (secondary care based) multidisciplinary team with relevant expertise at least in the first instance (then perhaps subsequent referral to a specialist tertiary team if deemed necessary)
- ◯ A woman is referred to a named link/specialist clinician/individual
- ◯ No formalized procedures in place or named link individuals. Referrals made on an ad-hoc basis.
- ◯ Other organisational model

Referral to a specialist team in a tertiary site

67. When was the specialist team first set up?

- ◯ Within the last 12 months
- ◯ 12-24 months ago
- ◯ Over 24 months ago
- ◯ Don't know

68. What is the membership of the specialist team? *Tick all that apply:*

- ☐ Diabetologist
- ☐ Obstetric Physician
- ☐ Other Physician (specify)
- ☐ Specialist diabetes midwife
- ☐ Woman's named midwife
- ☐ Other midwife
- ☐ Specialist diabetes nurse
- ☐ Obstetrician (ATSM or sub specialist trained in maternal medicine)
- ☐ Obstetrician
- ☐ Endocrinologist
- ☐ Neonatologist
- ☐ GP
- ☐ Anaesthetist
- ☐ Intensivist
- ☐ Dietician
- ☐ Not sure
- ☐ Other

If you selected other above please specify:

____

69. How does the team meet? *Tick all that apply*

- ☐ Within the clinic setting
- ☐ Separate to the clinic (e.g. in an MDT meeting)

70. Approximately how many women with pre-existing type 1 diabetes does the specialist team see for the first time per month?

- ◯ 1-2
- ◯ 3-5
- ◯ 6-10
- ◯ 11-20
- ◯ >20

71. Once pregnant, at what point during a woman's pregnancy is the first referral usually made to the MDT?

- ◯ First contact with health services when pregnant
- ◯ Booking visit (8-12 weeks)
- ◯ First scan (12 week scan)
- ◯ First hospital appointment with medical lead
- ◯ Anomaly scan (18-20 weeks)
- ◯ Other (please describe)

If you selected other above, please describe:

72. Do you have referral criteria in use for women with pre-existing type 1 diabetes?

- ◯ Yes
- ◯ No
- ◯ Don't know

73. Does regular evaluation of the outcomes of MDT care for women with pre-existing type 1 diabetes take place?

- ◯ Yes
- ◯ No
- ◯ Don't know

Referral to a local (secondary-care based) MDT

74. When was the team first set up?

- ◯ Within the last 12 months
- ◯ 12-24 months ago
- ◯ Over 24 months ago
- ◯ Don't know

75. What is the membership of the team? *Tick all that apply:*

- ☐ Diabetologist
- ☐ Obstetric Physician
- ☐ Other Physician (specify)
- ☐ Specialist diabetes midwife
- ☐ Woman's named midwife
- ☐ Other midwife
- ☐ Specialist diabetes nurse
- ☐ Obstetrician (ATSM or sub specialist trained in maternal medicine)
- ☐ Obstetrician
- ☐ Endocrinologist
- ☐ Neonatologist
- ☐ GP
- ☐ Anaesthetist
- ☐ Intensivist
- ☐ Dietician
- ☐ Not sure
- ☐ Other

If you selected other above please specify:

76. How does the team meet? *Tick all that apply*

- ☐ Within the clinic setting
- ☐ Separate to the clinic (e.g. in an MDT meeting)

77. Approximately how many women with pre-existing type 1 diabetes does the team see for thr first time per month?

- ◯ 1-2
- ◯ 3-5
- ◯ 6-10
- ◯ 11-20
- ◯ >20

78. Once pregnant, at what point during a woman's pregnancy is the first referral usually made to the team?

- ◯ First contact with health services when pregnant
- ◯ Booking visit (8-12 weeks)
- ◯ First scan (12 week scan)
- ◯ First hospital appointment with medical lead
- ◯ Anomaly scan (18-20 weeks)
- ◯ Other (please describe)

If you selected other above, please describe:

79. Do you have referral criteria in use for women with pre-existing type 1 diabetes?

- ◯ Yes
- ◯ No
- ◯ Don't know

80. Does regular evaluation of the outcomes of local MDT care for women with pre-existing type 1 diabetes take place?

- ◯ Yes
- ◯ No
- ◯ Don't know

81. Do you refer any women to a specialist MDT in a tertiary centre?

- ◯ Yes
- ◯ No

Referral to a specialist team in a tertiary site

82. When was the specialist team first set up?

- ◯ Within the last 12 months
- ◯ 12-24 months ago
- ◯ Over 24 months ago
- ◯ Don't know

83. What is the membership of the specialist team? *Tick all that apply:*

- ☐ Diabetologist
- ☐ Obstetric Physician
- ☐ Other Physician (specify)
- ☐ Specialist diabetes midwife
- ☐ Woman’s named midwife
- ☐ Other midwife
- ☐ Specialist diabetes nurse
- ☐ Obstetrician (ATSM or sub specialist trained in maternal medicine)
- ☐ Obstetrician
- ☐ Endocrinologist
- ☐ Neonatologist
- ☐ GP
- ☐ Anaesthetist
- ☐ Intensivist
- ☐ Dietician
- ☐ Not sure
- ☐ Other

If you selected other above please specify:

____

84. How does the team meet? *Tick all that apply*

- ☐ Within the clinic setting
- ☐ Separate to the clinic (e.g. in an MDT meeting)

85. Approximately how many women with pre-existing type 1 diabetes are referred to the specialist team for the first time per month?

- ◯ 1-2
- ◯ 3-5
- ◯ 6-10
- ◯ 11-20
- ◯ >20

86. Once pregnant, at what point during a woman's pregnancy is the first referral usually made to the MDT?

- ◯ First contact with health services when pregnant
- ◯ Booking visit (8-12 weeks)
- ◯ First scan (12 week scan)
- ◯ First hospital appointment with medical lead
- ◯ Anomaly scan (18-20 weeks)
- ◯ Other (please describe)

If you selected other above, please describe:

87. Do you have referral criteria in use for women with pre-existing type 1 diabetes?

- ◯ Yes
- ◯ No
- ◯ Don't know

88. Does regular evaluation of the outcomes of MDT care for women with pre-existing type 1 diabetes take place?

- ◯ Yes
- ◯ No
- ◯ Don't know

Referral to a named link/specialist clinician/individual

89. What is the professional group of the named link/specialist clinician/individual?

- ◯ Diabetologist
- ◯ Obstetric Physician
- ◯ Other Physician (specify)
- ◯ Specialist diabetes midwife
- ◯ Woman's named midwife
- ◯ Other midwife
- ◯ Specialist diabetes nurse
- ◯ Obstetrician (ATSM or sub specialist trained in maternal medicine)
- ◯ Obstetrician
- ◯ Endocrinologist
- ◯ Neonatologist
- ◯ GP
- ◯ Anaesthetist
- ◯ Intensivist
- ◯ Dietician
- ◯ Not sure
- ◯ Other

If you selected other above please specify:

90. Approximately how many women with pre-existing type 1 diabetes are referred to this link person for the first time <u>per month</u>?

- ◯ 1-2
- ◯ 3-5
- ◯ 6-10
- ◯ 11-20
- ◯ >20

91. Once pregnant, at what point during a woman's pregnancy is the first referral usually made to the link person?

- ◯ First contact with health services when pregnant
- ◯ Booking visit (8-12 weeks)
- ◯ First scan (12 week scan)
- ◯ First hospital appointment with medical lead
- ◯ Anomaly scan (18-20 weeks)
- ◯ Other (please describe)

If you selected other above, please describe:

No formalized procedures in place or named link individuals. Referrals mad...

92. Please describe how decisions about the management of women with pre-existing type 1 diabetes are usually made (in terms of the professionals involved and referral pathways, or variation in practice within your unit between different obstetricians etc).

Professionals involved and how they typically refer to each other:

Variation in practice within the unit (if applicable):

93. Approximately how many women with pre-existing type 1 diabetes are referred for the first time per month?

- ◯ 1-2
- ◯ 3-5
- ◯ 6-10
- ◯ 11-20
- ◯ >20

Other organisational model

94. Please describe how decisions about the management of women with congenital or acquired cardiac conditions are usually made (in terms of the professionals involved and referral pathways, or variation in practice within your unit between different obstetricians etc).

Professionals involved and how they typically refer to each other:

Variation in practice within the unit (if applicable):

95. Approximately how many women with congenital or acquired cardiac conditions are referred for the first time per month?

- ○ 1-2
- ○ 3-5
- ○ 6-10
- ○ 11-20
- ○ >20

96. Which of the following best describes the way that decisions are reached about the antenatal management of women with pre-existing Type 2 diabetes? Select the option that best reflects your current practice:

- ◯ A woman is referred directly to a specialist team in a tertiary centre: A multidisciplinary team of clinicians and midwives with different expertise who meet (either face to face or using videoconferencing) regularly to discuss individual cases –either in clinic or other setting.
- ◯ A woman is referred to a local (secondary care based) multidisciplinary team with relevant expertise at least in the first instance (then perhaps subsequent referral to a specialist tertiary team if deemed necessary)
- ◯ A woman is referred to a named link/specialist clinician/individual
- ◯ No formalized procedures in place or named link individuals. Referrals made on an ad-hoc basis.
- ◯ Other organisational model

Referral to a specialist team in a tertiary site

97. When was the specialist team first set up?

- ◯ Within the last 12 months
- ◯ 12-24 months ago
- ◯ Over 24 months ago
- ◯ Don't know

98. What is the membership of the specialist team? *Tick all that apply:*

- ☐ Diabetologist
- ☐ Obstetric Physician
- ☐ Other Physician (specify)
- ☐ Specialist diabetes midwife
- ☐ Woman's named midwife
- ☐ Other midwife
- ☐ Specialist diabetes nurse
- ☐ Obstetrician (ATSM or sub specialist trained in maternal medicine)
- ☐ Obstetrician
- ☐ Endocrinologist
- ☐ Neonatologist
- ☐ GP
- ☐ Anaesthetist
- ☐ Intensivist
- ☐ Dietician
- ☐ Not sure
- ☐ Other

If you selected other above please specify:

99. How does the team meet? *Tick all that apply*

- ☐ Within the clinic setting
- ☐ Separate to the clinic (e.g. in an MDT meeting)

100. Approximately how many women with pre-existing type 2 diabetes does the specialist team see for the first time per month?

- ◯ 1-2
- ◯ 3-5
- ◯ 6-10
- ◯ 11-20
- ◯ >20

101. Once pregnant, at what point during a woman's pregnancy is the first referral usually made to the MDT?

- ◯ First contact with health services when pregnant
- ◯ Booking visit (8-12 weeks)
- ◯ First scan (12 week scan)
- ◯ First hospital appointment with medical lead
- ◯ Anomaly scan (18-20 weeks)
- ◯ Other (please describe)

If you selected other above, please describe:

102. Do you have referral criteria in use for women with pre-existing type 2 diabetes?

- ◯ Yes
- ◯ No
- ◯ Don't know

103. Does regular evaluation of the outcomes of MDT care for women with pre-existing type 2 diabetes take place?

- ◯ Yes
- ◯ No
- ◯ Don't know

Referral to a local (secondary-care based) MDT

104. When was the team first set up?

- ◯ Within the last 12 months
- ◯ 12-24 months ago
- ◯ Over 24 months ago
- ◯ Don't know

105. What is the membership of the team? *Tick all that apply:*

- ☐ Diabetologist
- ☐ Obstetric Physician
- ☐ Other Physician (specify)
- ☐ Specialist diabetes midwife
- ☐ Woman's named midwife
- ☐ Other midwife
- ☐ Specialist diabetes nurse
- ☐ Obstetrician (ATSM or sub specialist trained in maternal medicine)
- ☐ Obstetrician
- ☐ Endocrinologist
- ☐ Neonatologist
- ☐ GP
- ☐ Anaesthetist
- ☐ Intensivist
- ☐ Dietician
- ☐ Not sure
- ☐ Other

If you selected other above please specify:

106. How does the team meet? *Tick all that apply*

- ☐ Within the clinic setting
- ☐ Separate to the clinic (e.g. in an MDT meeting)

107. Approximately how many women with pre-existing type 2 diabetes does the team see for the first time per month?

- ◯ 1-2
- ◯ 3-5
- ◯ 6-10
- ◯ 11-20
- ◯ >20

108. Once pregnant, at what point during a woman's pregnancy is the first referral usually made to the team?

- ◯ First contact with health services when pregnant
- ◯ Booking visit (8-12 weeks)
- ◯ First scan (12 week scan)
- ◯ First hospital appointment with medical lead
- ◯ Anomaly scan (18-20 weeks)
- ◯ Other (please describe)

If you selected other above, please describe:

109. Do you have referral criteria in use for women with pre-existing type 2 diabetes?

- ◯ Yes
- ◯ No
- ◯ Don't know

110. Does regular evaluation of the outcomes of local MDT care for women with pre-existing type 2 diabetes take place?

- ◯ Yes
- ◯ No
- ◯ Don't know

111. Do you refer any women to a specialist MDT in a tertiary centre?

- ◯ Yes
- ◯ No

Referral to a specialist team in a tertiary site

112. When was the specialist team first set up?

- ◯ Within the last 12 months
- ◯ 12-24 months ago
- ◯ Over 24 months ago
- ◯ Don't know

113. What is the membership of the specialist team? *Tick all that apply:*

- ☐ Diabetologist
- ☐ Obstetric Physician
- ☐ Other Physician (specify)
- ☐ Specialist diabetes midwife
- ☐ Woman's named midwife
- ☐ Other midwife
- ☐ Specialist diabetes nurse
- ☐ Obstetrician (ATSM or sub specialist trained in maternal medicine)
- ☐ Obstetrician
- ☐ Endocrinologist
- ☐ Neonatologist
- ☐ GP
- ☐ Anaesthetist
- ☐ Intensivist
- ☐ Dietician
- ☐ Not sure
- ☐ Other

If you selected other above please specify:

114. How does the team meet? *Tick all that apply*

- ☐ Within the clinic setting
- ☐ Separate to the clinic (e.g. in an MDT meeting)

115. Approximately how many women with pre-existing type 2 diabetes are referred to the specialist team for the first time per month?

- ◯ 1-2
- ◯ 3-5
- ◯ 6-10
- ◯ 11-20
- ◯ >20

116. Once pregnant, at what point during a woman's pregnancy is the first referral usually made to the MDT?

- ◯ First contact with health services when pregnant
- ◯ Booking visit (8-12 weeks)
- ◯ First scan (12 week scan)
- ◯ First hospital appointment with medical lead
- ◯ Anomaly scan (18-20 weeks)
- ◯ Other (please describe)

If you selected other above, please describe:

117. Do you have referral criteria in use for women with pre-existing type 2 diabetes?

- ◯ Yes
- ◯ No
- ◯ Don't know

118. Does regular evaluation of the outcomes of MDT care for women with pre-existing type 2 diabetes take place?

- ◯ Yes
- ◯ No
- ◯ Don't know

Referral to a named link/specialist clinician/individual

119. What is the professional group of the named link/specialist clinician/individual?

- Diabetologist
- Obstetric Physician
- Other Physician (specify)
- Specialist diabetes midwife
- Woman's named midwife
- Other midwife
- Specialist diabetes nurse
- Obstetrician (ATSM or sub specialist trained in maternal medicine)
- Obstetrician
- Endocrinologist
- Neonatologist
- GP
- Anaesthetist
- Intensivist
- Dietician
- Not sure
- Other

If you selected other above please specify:

120. Approximately how many women with pre-existing type 2 diabetes are referred to this link person for the first time per month?

- 1-2
- 3-5
- 6-10
- 11-20
- >20

121. Once pregnant, at what point during a woman's pregnancy is the first referral usually made to the link person?

- ◯ First contact with health services when pregnant
- ◯ Booking visit (8-12 weeks)
- ◯ First scan (12 week scan)
- ◯ First hospital appointment with medical lead
- ◯ Anomaly scan (18-20 weeks)
- ◯ Other (please describe)

If you selected other above, please describe:

122. Please describe how decisions about the management of women with pre-existing type 2 diabetes are usually made (in terms of the professionals involved and referral pathways, or variation in practice within your unit between different obstetricians etc).

Professionals involved and how they typically refer to each other: ______

Variation in practice within the unit (if applicable): ______

123. Approximately how many women with pre-existing type 2 diabetes are referred for the first time <u>per month</u>?

- ◯ 1-2
- ◯ 3-5
- ◯ 6-10
- ◯ 11-20
- ◯ >20

Other organisational model

124. Please describe how decisions about the management of women with congenital or acquired cardiac conditions are usually made (in terms of the professionals involved and referral pathways, or variation in practice within your unit between different obstetricians etc).

Professionals involved and how they typically refer to each other:

Variation in practice within the unit (if applicable):

125. Approximately how many women with congenital or acquired cardiac conditions are referred for the first time per month?

- ○ 1-2
- ○ 3-5
- ○ 6-10
- ○ 11-20
- ○ >20

Thank You

126. Thank you for participating in this survey.

If you want to be involved in this research and/or would be willing to speak to us further about the organisational management of women with high risk conditions please enter your details below and we will contact you to arrange a mutually convenient time to speak/meet:

Name:

Email address:

Telephone number:

To contact us directly please email Cath.Taylor@kcl.ac.uk
